# Complete Mitochondrial Genome of a French Bulldog with *Carcinoma planoepitheliale keratodes G1*: A Descriptive Case Report

**DOI:** 10.3390/genes17101203

**Published:** 2026-09-29

**Authors:** Krzysztof Kowal, Kaja Ziółkowska-Twarowska, Bartłomiej Kiecak, Norbert Ozga, Angelika Tkaczyk-Wlizło, Paweł Grychnik, Ludmiła Grzybowska-Szatkowska, Brygida Ślaska

**Affiliations:** 1 Institute of Biological Basis of Animal Production, University of Life Sciences in Lublin, Akademicka 13 St., 20-950 Lublin, Poland; 2 Department of Radiotherapy, Medical University of Lublin, Chodźki 7, 20-093 Lublin, Poland

**Keywords:** French Bulldog, mitochondrial genome, sequence variants

## Abstract

French Bulldogs, like other brachycephalic breeds, are predisposed to a variety of genetic and tumour-related disorders. While mitochondrial DNA (mtDNA) mutations have been documented in canine tumours, no prior study has examined the entire mitochondrial genome in this breed. In this study, we analysed mtDNA obtained from blood and tumour tissue of a 10-year-old male French Bulldog diagnosed with *carcinoma planoepitheliale keratodes* (G1). Complete sequencing and comparative bioinformatic analysis revealed 22 sequence variants, including nine in protein-coding genes, two in RNA-coding genes, two in the control region, and nine within the variable number tandem repeat (VNTR) region. Identical variants were present in both blood and tumour tissue, indicating that no detectable tumour-specific mtDNA mutations were observed. Notably, the m.1397A>T transversion in the 16S rRNA gene and an insertion (m.2679_2680insG) in the tRNA-Leu(UUR) gene were identified, both mapping to conserved loci in the human mitochondrial genome. Among the coding variants, four nonsynonymous variants (including p.Cys55Tyr and p.Ser471Asn) were detected, two of which affected highly conserved amino acid positions. Most nonsynonymous changes were localised in random coil regions and internal mitochondrial compartments, whereas synonymous changes appeared mainly within transmembrane helices. The control region displayed the highest degree of polymorphism, including eight positions with A/G heteroplasmy and one length variant due to polyT extension. These findings represent the first documentation of full mitochondrial genome sequence variants in a French Bulldog with cancer providing descriptive data for future breed population and oncological research. Further studies are warranted to determine whether these alterations are breed- or tumour-specific.

## 1. Introduction

French Bulldogs share several key genetic mutations and traits with other small brachycephalic breeds such as Bulldogs and Boston Terriers, but there are also breed-specific differences in mutation prevalence and diversity [1]. This breed has a higher risk for various disorders often related to its brachycephalic conformation. These include ocular, respiratory, neurological, and dermatological problems [2]. Genetic studies have identified nuclear gene variants associated with significant health problems in this breed may be present, such as muscle hypertrophy, dysphagia, and gait abnormalities [3]. Notably, variants were found in the *CLCN1* gene (chloride channel gene) causing non-dystrophic congenital myotonia and in the *PDE4C* gene (phosphodiesterase gene) that affects skeletal muscle function [3]. French Bulldogs tend to have slightly higher genetic diversity for many *loci*, suggesting more genetic variation within the breed. This is important as it affects the potential for breed-specific health issues and the ability to manage these via breeding strategies [4].

Current studies regarding molecular changes in French Bulldogs tend to focus on mutations in the nuclear genome (nDNA). Mitochondria play a central role in cellular bioenergetics, metabolic regulation and apoptosis [5,6]. In canine oncology, alterations within mtDNA, predominantly investigated in targeted regions such as the displacement loop (D-loop) or genes encoding respiratory-chain subunits, have been documented in various tumour types [7]. These studies suggest that the alterations in mtDNA might occur in dogs in various pathological states, but do not provide breed-specific findings for French Bulldog. However, the majority of earlier canine studies sequenced only limited fragments of mtDNA, leaving potential variations across the full mitogenome uncharacterised [8,9,10]. Furthermore, complete mitogenome sequences specifically derived from French Bulldogs remain largely underrepresented in public genomic repositories. To date, there is no direct study describing mtDNA variants or changes exclusively in this breed.

The aim of this case study was to characterise the complete mitochondrial genome in both tumour tissue and a matched blood sample from a 10-year-old French Bulldog with *carcinoma planoepitheliale keratodes* G1.

## 2. Materials and Methods

The entire mitochondrial genome, isolated from two tissues—blood (healthy tissue) and tumour—obtained from an individual 10-year-old male French Bulldog, was analysed. The dog had a malignant *carcinoma planoepitheliale keratodes* G1 (low-grade) tumour localised on a paw. The tumour tissue sample was excised and then placed in a sterile container. Blood was sampled into sterile tubes with the K_3_EDTA (Greiner Bio-One, Kremsmünster, Austria) anticoagulant.

The tumour tissue sample was subjected to histopathology analysis. The samples were fixed in buffered formalin (Sigma-Aldrich, Darmstadt, Germany), pH 7.2, processed routinely, embedded in paraffin wax, sectioned into 4 μm-thick sections, and stained with haematoxylin-eosin (Sigma-Aldrich, Darmstadt, Germany).

DNA was isolated from post-operative tumour tissue and blood sample with the DNeasy Blood & Tissue Kit (Qiagen, Hilden, Germany). DNA samples were assessed quantitatively and qualitatively by electrophoretic separation in 1% agarose gel (Bio-Rad Laboratories, Hercules, CA, USA) and spectrophotometrically (NanoDrop One, Thermo Fisher Scientific, Waltham, MA, USA). Selective amplification of mtDNA was performed on the total genomic DNA using two pairs of primers [11]. Full coverage of mtDNA was obtained after amplification of two ~10 kb polymerase chain reaction (PCR) products. Amplification reactions were carried out using KAPA HiFi PCR Kit reagents (KAPA Biosystems, Wilmington, MA, USA). The PCR products were analysed subsequently in 0.7% agarose gel and purified using AMPure XP magnetic beads (Beckman Coulter, Brea, CA, USA). DNA was quantified using a Qubit 3.0 fluorimeter (Thermo Fisher Scientific, Waltham, MA, USA). Both PCR products were diluted to the desired concentration and pooled in an equimolar ratio. The pooled DNA was then used for library preparation.

Mitochondrial DNA was sequenced in the DNA Sequencing and Oligonucleotide Synthesis Laboratory (Institute of Biochemistry and Biophysics, Polish Academy of Sciences). Approximately 1 ng of the PCR DNA template mix was used as an input, and an Illumina shotgun library was constructed using the Nextera XT Kit (Illumina, San Diego, CA, USA) following the manufacturer’s instructions. The library sample was sequenced on an Illumina MiSeq sequencer using a 600-cycle kit (v3) in paired-end mode targeting at least 100× coverage. The sequence reads were filtered by quality (Q > 30), and remaining adaptors were removed using the fastp tool (v. 1.3.7.) [12]. Cleaned sequencing reads were mapped to the dog mtDNA reference sequence (GenBank accession number: U96639.2) [13] using the Burrows–Wheeler Aligner (v. 0.7.19.) [14] and consensus sequences for each sample were generated using samtools and bcftools (v. 1.21.1.) [15]. Sequencing coverage statistics were calculated using the mosdepth tool (v. 0.3.11.) [16].

The obtained nucleotide sequences were subjected to bioinformatics analyses in order to determine mutation and polymorphic sites within the analysed mtDNA genome in the tumour tissue Unipro UGENE (v. 52.0) [17]. The differences in the nucleotide sequence were determined by comparison of the analysed sequences with the reference sequence [13]. Polymorphisms were defined as sequence changes relative to the reference sequence that were present in both tumour and blood cells. Mutations were defined as sequence changes characteristic of mtDNA from tumour cells but absent from mtDNA from the corresponding blood sample. The secondary structure protein analysis, the determination of transmembrane helices as well as the localisation of buried/exposed residues was performed with the Canis mitoSNP tool [18] which integrates predictions from SOPMA (secondary structure), TMHMM (transmembrane helices) and ConSurf (conservation grade).

To enable comparison between the canine and human mitochondrial genomes, the identified variant positions were mapped to their homologous sites in the human mitochondrial genome using the Canis mitoSNP tool, which references the revised human mtDNA sequence NC_012920.1 [19]. This cross-species mapping was performed because functional and clinical annotations of mitochondrial variants are considerably more comprehensive for the human mitogenome than for the canine mitogenome. Referring canine variants to homologous human positions therefore supports translational interpretation and allows the potential functional impact of canine variants to be inferred from existing human data.

The HGVS nomenclature was used for description of sequence variants found in the DNA and proteins [20].

## 3. Results

The comparative analysis of changes in the mitochondrial DNA sequences obtained from blood- and tumour-specific samples revealed 22 sequence variants relative to the reference sequence, of which two were located in RNA-coding genes, nine in protein-coding genes, two in the control region and nine in the VNTR region localised in the non-coding part of the mitochondrial genome. There were no differences between the healthy and tumour tissue in French Bulldog samples. No tumour-specific variants were observed. However, in the VNTR region, a heteroplasmy at eight positions was detected.

Bioinformatic analysis of sequences obtained from blood and tumour tissue revealed a transversion at position m.1397A>T, localised within the 16S rRNA gene. This transversion caused a change in the region of unpaired bases in the secondary structure of the molecule. The position m.1397A corresponded to a position m.1972A in the human mitochondrial DNA (Table 1). An indel variant was found in m.2679_2680insG—*MT-TL1 (UUR)* gene causing insertion in the DHU arm of the tRNA leucine molecule. The position m.2679G corresponded to a position m.3239G in the human mitochondrial DNA, which interestingly was placed in *MT-TL1* gene and *MT-TER* (mitochondrial transcription termination region) region (Table 1).

Among the nine alterations identified in protein-coding genes, seven transitions, one duplication and one transversion were observed. Four of the nine polymorphisms caused nonsynonymous amino acid substitutions, two of which (p.Cys55Tyr, p.Ser471Asn) were classified as nonconservative replacements (Table 2). Amino acid substitutions p.Met1Val and p.Ser508Thr were classified as conservative replacements and p.Cys55Tyr and p.Ser471Asn as nonconservative replacements. Among all nine variants, five were localised in the random coil secondary structure of a protein. Nonsynonymous changes were mainly localised in the internal part of the mitochondrion, whereas synonymous changes were mainly in the transmembrane helix. The variant p.Leu135= was classified as a change in structural residue, whereas p.Ser508Thr variant was a functional residue. Most of the alterations (6/9) occurred in buried residues of a protein. Two variants caused alterations in highly conserved amino acid positions: p.Ser508Thr and p.Met1Val (Table 2).

The control region of the dog’s mitochondrial DNA was the most variable region in the whole genome. Among eleven variants, nine were placed in the VNTR region and eight of them were A/G or G/A heteroplasmy, whereas only m.16338G>A was homoplasmic in both samples (Table 3). There was a length variant observed in the control region as well, which was an extension of a homopolymer polyT. Based on the quality of the variant call and its consistent detection in both independently sequenced blood and tumour samples, we excluded the explanation of a homopolymer sequencing artifact as the cause of this length variant.

## 4. Discussion

To the best of the authors’ knowledge, this is the first report describing molecular alterations in the whole mitochondrial DNA in a case of a French Bulldog diagnosed with a tumour. Previous canine studies presented some changes in the D-loop [21] or protein-coding genes [22] in various dog breeds including French Bulldogs. However, it should be emphasised that this research did not fully elucidate potential changes to the mitochondrial genome.

The identified variants with specific variants corresponding to homologous sites in the human mitochondrial genome were presented to broaden the plausible implications of changes in these regions. Of particular interest was the m.1397A>T transversion, localised within the 16S rRNA gene, altering the secondary structure of unpaired bases. This site aligns with m.1972A in human mtDNA. It should be stated that this variant is described for the first time in the literature regarding molecular changes in French Bulldogs. In another study conducted by the authors, frequent variants were found in m.1351A>G and m.1454G>A [23]. Similarly, the substitution m.13189G>A in the *MT-ND5* gene has never been described in any other cases of dogs with tumours. However, it should be noted that these two variants were identified in several canine population studies on mtDNA haplotypes [11,24,25], therefore may represent breed-typical rather than tumour-associated variants.

Ślaska et al. (2015) identified some variants in *MT-ND1* (216C>T corresponded to m.2962C>T variant) and *MT-CO1* (507C>T corresponded to m.5855C>T variant) genes in the case of a 4-year-old male French Bulldog with *lymphoma centroblasticum* [22]. Interestingly, these variants did not cause a change in the amino acid sequence. The same dog did not display any mutations or polymorphisms in the D-loop region [26]. Surdyka and Ślaska (2017) [9,21] analysed the mtDNA regions in the case of an 8-year-old French Bulldog with a mammary gland tumour, *adenoma G2.* The results revealed two more frequently observed variants, m.15435T>C (frequency = 0.81) and m.15814C>T (frequency = 0.94), in the D-loop region and two variants in protein-coding regions: m.4303T>C (*MT-ND2*, frequency = 0.16) and m.8313delA (*MT-ATP6*, frequency = 1.00) [9,21]. In our case we did not observe any deletions in the analysed samples; however, it is difficult to indicate that this polymorphism is more specific for tumours.

The nonsynonymous polymorphisms detected in this study (Table 2) differed in their predicted structural impact. While p.Met1Val and p.Ser508Thr were classified as conservative replacements, the other two variants, p.Cys55Tyr and p.Ser471Asn, were determined to be nonconservative, potentially affecting protein conformation and function. Notably, p.Cys55Tyr involved a sulphur-containing residue substitution, which may have structural consequences in the local protein fold.

Most of the variants were positioned within the random coil regions of the secondary structure. The nonsynonymous substitutions were primarily localised in the internal compartment of the mitochondrion, a site associated with matrix-facing enzymatic activities, while synonymous variants were mainly embedded within the transmembrane helices, which could suggest a possible selective constraint acting on surface-exposed residues. Furthermore, six out of nine changes were found in buried residues, implying potential stability-related roles.

Interestingly, the two variants occurring at highly conserved positions (p.Ser508Thr, p.Met1Val; Table 2) merit closer consideration. However, a p.Met1Val variant was observed in almost all cases of analysed dogs in our previous studies regardless of their breed [10,23,27]. The universal occurrence of p.Met1Val across breeds in our previous studies suggests that it reflects a feature of the reference sequence rather than a biologically meaningful change.

The control region of the mitochondrial genome, particularly the VNTR segment, exhibited the highest variability among all examined regions. This observation aligns with previous findings indicating the D-loop as a mutation hotspot, particularly in dogs diagnosed with mammary or epithelial tumours [21]. In the current case, the changes in the VNTR region were predominantly transitions, consistent with the known mutational patterns in the non-coding mitochondrial genome.

Length variant, observed in this study as a duplication polymorphism extending a homopolymeric polyT tract, has also been reported previously in canine mtDNA and is considered a common form of instability within the mitochondrial genome [27] rather than the artifact of the sequencing method [28]. It has been hypothesised that slippage events during replication may contribute to this phenomenon, especially in mononucleotide tracts, which are prone to polymerase errors [29]. Although the functional impact of these changes remains unclear, it is possible that they may alter the replication origin efficiency or affect transcription factor binding sites, particularly in polymorphic motifs situated near the conserved sequence blocks (CSBs).

Moreover, in one of the positions within the VNTR region, a complete G>A transition was observed. Unlike the heteroplasmic sites, this substitution was homoplasmic and could represent a stabilised polymorphism. Its detection in both healthy and tumour-derived DNA may indicate either an inherited variant or an early somatic event fixed during clonal expansion of the tumour. However, based on the nature of this study, the explanation requires further research.

In contrast to protein-coding or RNA genes, variants in the control region cannot be directly linked with amino acid changes or structural disruptions. This region of the mitogenome is responsible for DNA replication and transcription [30,31,32]. However, the increasing number of studies, including those focusing solely on the D-loop, suggest that accumulation of polymorphisms in this region may serve as a molecular signature of tumour development or progression in dogs [10]. Whether these variants are causative or reflective of mitochondrial dysfunction remains speculative.

Although a comprehensive analysis of the mitochondrial genome was performed, the presented results are based on a single individual, which inherently limits broader generalisations. Despite the high number of identified polymorphisms and structural alterations, it cannot be conclusively determined whether these changes are causative, consequential, or incidental in the context of neoplastic transformation. For future research, extension of this approach to a larger cohort of French Bulldogs, both healthy and tumour-bearing, is recommended. Comparative studies across different breeds and tumour types could also help establish whether certain polymorphisms are breed-specific, tumour-specific, or universal markers of mitochondrial instability.

## 5. Conclusions

This study constitutes the first comprehensive analysis of the entire mitochondrial genome in a French Bulldog diagnosed with *carcinoma planoepitheliale keratodes*. The identification of 22 sequence variants, including rare and potentially impactful changes in rRNA, tRNA, and protein-coding genes, highlights a plausible molecular complexity of mtDNA in this breed, yet the actual impact should be confirmed on a larger cohort. Particularly noteworthy are the m.1397A>T transversion in the 16S rRNA gene and the insertion in *MT-TL1(UUR)*, both located in structurally significant regions of the mitochondrial transcriptome. Additionally, nonsynonymous variants such as p.Cys55Tyr and p.Ser471Asn may suggest potential consequences for mitochondrial protein stability and function. The control region, especially the VNTR segment, showed substantial variability, including frequent heteroplasmic transitions and a duplication polymorphism within a homopolymeric tract. These observations are consistent with previous reports describing the control region as the most variable segment of the canine mitochondrial genome. Because all variants were also present in blood, the present data do not support a tumour-specific origin.

Despite its single-case design, this study expands current knowledge on mitochondrial polymorphisms in tumour-bearing French Bulldogs and introduces potentially breed-specific findings that have not been previously described.

## Figures and Tables

**Table 1:** Variants in the RNA-coding genes observed in tumour and blood samples compared with the reference sequence.

Position	Tumour	Blood	Variant	Gene	tRNA/rRNA Secondary Structure	Human mtDNA Position
m.1397A	T	T	m.1397A>T	16S rRNA	unpaired bases	m.1972A
m.2679_2680	G	G	m.2679_2680insG	*MT-TL1 (UUR)*	DHU arm	m.3239G

**Table 2:** Variants in the protein-coding genes observed in tumour and blood samples compared with the reference sequence.

Gene	Position	Variant	Codon Change	Amino-Acid Variant	SOPMA	TMHMM	CG *	Buried/ Exposed Residue	Human mtDNA Position
*MT-CO1*	m.5367C	m.5367C>T	CTG → TTG	p.Leu7=	Extended strand	outside	2	buried	m.5922C
	m.5444T	m.5444T>C	GCT → GCC	p.Ala32=	Alpha helix	TMhelix	1	buried	m.5999T
	m.6065A	m.6065A>G	GGA → GGG	p.Gly239=	Random coil	TMhelix	9	buried	m.6620T
*MT-ATP6*	m.8368C	m.8368C>T	CTC → CTT	p.Leu135=	Random coil	TMhelix	1	exposed ^2^	m.8931A
*MT-CO3*	m.8807G	m.8807G>A	TGC → TAC	p.Cys55Tyr	Alpha helix	inside	1	buried	m.9370A
*MT-ND4L*	m.9912_9913TG	m.9912_9913dup	ATG → GTG	p.Met1Val	Random coil	outside	8	exposed	m.10472G
*MT-ND5*	m.11939C	m.11939C>T	TTC → TTT	p.Phe54=	Extended strand	inside	4	buried	m.12498C
	m.13189G	m.13189G>A	AGC → AAC	p.Ser471Asn	Random coil	inside	3	buried	m.13748A
	m.13299T	m.13299T>A	TCA → ACA	p.Ser508Thr	Random coil	inside	6	exposed ^3^	m.13858A

* Conservation grade: from 1 to 4—variable, from 5 to 9—conserved, ^2^—structural residue, ^3^—functional residue, SOPMA, Self-Optimized Prediction Method with Alignment; TMHMM, Transmembrane Hidden Markov Model.

**Table 3:** Variants in the non-coding region observed in tumour and blood samples compared with the reference sequence. R denotes A/G (adenine/guanine) in the IUPAC nucleotide code.

Position	Tumour	Blood	Variant	Region
m.15814C	T	T	m.15814C>T	control region
m.16148A	R	R	m.16148A/G	VNTR
m.16168A	R	R	m.16168A/G	VNTR
m.16178A	R	R	m.16178A/G	VNTR
m.16188G	R	R	m.16188G/A	VNTR
m.16208A	R	R	m.16208A/G	VNTR
m.16228G	R	R	m.16228G/A	VNTR
m.16338G	A	A	m.16338G>A	VNTR
m.16378G	R	R	m.16378G/A	VNTR
m.16398A	R	R	m.16398A/G	VNTR
m.16671T	T	T	m.16671_16672dup	control region

## Data Availability

The data obtained after NGS sequencing generated in this study have been submitted to the NCBI BioProject database under accession number PRJNA1452610.

## Abbreviations

The following abbreviations are used in this manuscript:

16S rRNA	16 Svedberg ribosomal ribonucleic acid
BWA	Burrows–Wheeler Aligner
CG	Conservation grade
CLCN1	Chloride voltage-gated channel 1 gene
D-loop	Displacement loop
DHU	Dihydrouridine
DNA	Deoxyribonucleic acid
HGVS	Human Genome Variation Society
IUPAC	International Union of Pure and Applied Chemistry
MT-ATP6	Mitochondrially encoded ATP synthase membrane subunit 6 gene
MT-CO1/3	Mitochondrially encoded cytochrome c oxidase subunit 1/3 gene
MT-ND1/5	Mitochondrially encoded NADH dehydrogenase subunit 1/5 gene
MT-ND4L	Mitochondrially encoded NADH:ubiquinone oxidoreductase core subunit 4L gene
MT-TER	Mitochondrially encoded transcription terminator gene
MT-TL1	Mitochondrially encoded tRNA leucine (UUR) gene
mtDNA	Mitochondrial deoxyribonucleic acid
NCBI	National Center for Biotechnology Information
nDNA	Nuclear deoxyribonucleic acid
NGS	Next-Generation Sequencing
PCR	Polymerase chain reaction
PDE4C	Phosphodiesterase 4C gene
RNA	Ribonucleic acid
SOPMA	Self-Optimized Prediction Method with Alignment
TMHMM	Transmembrane Hidden Markov Model
tRNA	Transfer ribonucleic acid
VNTR	Variable number tandem repeat
